# Pseudotumors and tumors of the temporomandibular joint. A review

**DOI:** 10.4317/medoral.18799

**Published:** 2013-02-05

**Authors:** Rafael Poveda-Roda, José V. Bagán, José M. Sanchis, María Margaix

**Affiliations:** 1 MD, DDS, PhD. Department of Stomatology and Maxillofacial Surgery. Valencia University General Hospital; 2 MD, DDS, PhD. Head of the Department of Stomatology and Maxillofacial Surgery. Chairman of Oral Medicine. University of Valencia; 3 DDS, PhD. Associate Professor of Oral Medicine. Department of Stomatology, University of Valencia. Valencia (Spain)

## Abstract

Objective: To review the pseudotumors and tumors of the temporomandibular joint (TMJ) published in journals included in Journal Citation Reports (JCR), and to evaluate whether there are clinical and radiological signs capable of differentiating between pseudotumors and tumors and between malignant and benign tumors. Material and Methods: A systematic Medline search was made of clinical cases of tumors and pseudotumors of the TMJ covering a 20-year period and published in journals included in JCR. Only cases with histological confirmation were included. A description is provided of the general characteristics of TMJ tumors, with comparison of the clinical, diagnostic, therapeutic and evolutive variables referred to pseudotumors, benign tumors and malignant tumors. Results: We identified 285 TMJ tumors published in 181 articles of 15 journals. The most frequent lesions were pseudotumors (synovial chondromatosis, pigmented villonodular synovitis, eosinophilic granuloma and osteochondroma). The mean age was 42 years and one month ± 16 years and two months. Tumors were more common in females. The mean time from symptoms onset to consultation was 30 months and 8 days ± 41 months and 9 days, and almost 19.6% of the cases initially had been diagnosed and treated as TMJ dysfunction. The most frequent clinical manifestations were pain, swelling and the limitation of joint movements. The most common radiological findings in the case of benign and malignant lesions were radiopacities and radiotransparencies, respectively. No panoramic X-ray alterations were observed in 14.6% of the benign tumors and in 7.7% of the malignant lesions. Surgery was the usual form of treatment. Sequelae were recorded in 18.2% of the cases, with tumor relapse in 9.1%. The four-year survival rate in the case of malignant tumors was 72.2%.

** Key words:**Tumor, temporomandibular joint, metaplasia, pseudotumor, condyle.

## Introduction

Temporomandibular joint (TMJ) disorders, also generically referred to as TMJ dysfunction or derangement (TMJD), are relatively common (1) and constitute the leading cause of orofacial pain of non-dental origin (2). Most of these disorders are benign and tend to resolve spontaneously (3). TMJ pseudotumors and tumors are infrequent (4), and clinically manifest in a way very similar to TMJD (5). Certain clinical manifestations can help us distinguish between TMJD and tumor disease: numbness of the territories innervated by the trigeminal nerve branches, hearing loss, constant pain not influenced by mandibular movements, increased severity of symptoms, a lack of response to treatment, alterations in dental occlusion, the presence of swelling (including adenopathies), unexplained weight loss, ear suppuration or swallowing difficulties (6). However, these manifestations are not present in all cases, and many patients with TMJ tumors are initially diagnosed and treated as cases of TMJD (7). The resulting delay in establishing the correct diagnosis causes increased suffering, a greater risk of treatment complications and, in the case of malignant tumors, an increased threat to patient survival. In this context it is necessary to identify the symptoms, signs and radiological alterations advising the inclusion of TMJ tumors in the differential diagnosis (8).

There are a number of TMJ lesions that are characterized by tissue growth, but which have not been clearly established as true tumors (9-11) (osteochondroma, pigmented villonodular synovitis, eosinophilic granuloma). There is widespread agreement that synovial chondromatosis corresponds to metaplastic transformation of synovial tissue into chondroid tissue. Independently of the debate as to whether these are genuine tumors or not, we have included them as pseudotumors in this review.

The present study reviews the TMJ pseudotumors and tumors published over the last 20 years in journals included in *Journal Citation Reports* (JCR) under the category *Dentistry, Oral Surgery and Medicine*, and an evaluation is made of whether there are clinical and radiological signs capable of differentiating between pseudotumors and tumors and between malignant and benign tumors of the TMJ, with tabulation of the clinical, therapeutic and prognostic findings of the most common TMJ neoplasms.

## Material and Methods

A list was obtained of the journals included in JCR under the category *Dentistry, Oral Surgery and Medicine* up until December 2009 (56 journals).

A Medline search was made using the following terms and boolean operators: *Temporomandibular AND tumor, temporomandibular and pseudotumor, condyle AND tumor, condyle AND pseudotumor, synovial chondromatosis AND temporomandibular, osteocartilaginous exostosis AND temporomandibular, eosinophilic granuloma AND temporomandibular, pigmented synovitis AND temporomandibular*. The search was limited to a time period of 20 years (from 1 January 1990 to 31 December 2009), and included only case reports and case series.

From the list of articles thus obtained, a manual selection was made of those papers containing descriptions of clinical cases of TMJ pseudotumors and tumors published in journals included in the JCR under the category *Dentistry, Oral Surgery and Medicine*. All included cases were required to have histological confirmation of the diagnosis. Three articles were excluded due to a lack of histological confirmation. The only exception to this requirement was the presence of metastases, in which a Department of Oncology confirmed the TMJ lesion as representing the metastatic expression of malignant disease.

The disease processes included in the study under the category *pseudotumors* were osteochondroma, synovial chondromatosis, pigmented villonodular synovitis and eosinophilic granuloma. In the present study the term “benign lesions” is used in joint reference to pseudotumors and benign tumors.

Pseudotumors and tumors of the mandibular coronoid process were not included in the study.

A descriptive statistical study was made, expressing qualitative variables as absolute and relative frequencies, and quantitative data as the mean and standard deviation. The comparative analysis was based on the chi-squared test and analysis of variance (ANOVA). Survival analysis in turn was based on the Kaplan-Meier test and the Mantel-Cox log-rank test.

Dichotomic clinical manifestations and the imaging study data were subjected to diagnostic validity testing (sensitivity (Se), specificity (Sp), positive predictive value (PPV) and negative predictive value (NPV)), though only those yielding values of over 90% have been included in the text.

In  Table 1 Chi-square test is used to analyze differences in clinical variables between tumors and pseudotumors and between benign and malignant tumors. For  Table 2, Table 3, Table 4 three quality levels have been established according to the number of cases included in the publication and the number of cases offering information referred to a concrete study variable. The data of adequate quality (over 20 cases and information referred to a concrete variable available in over 66% of the cases) appear in the tables over a dark gray background. Medium quality data (over 20 cases and information available in 33-66% of the cases, or between 10-20 cases and information available in over 66% of the cases) in turn appear over a light gray background, while limited quality data (fewer than 10 cases and information available in under 33% of the cases, or between 10-20 cases and information available in under 66% of the cases) appear over a white background. This simply constitutes an *ad hoc* system for classifying the quality of the clinical information contained in the articles.

**Table 1:**
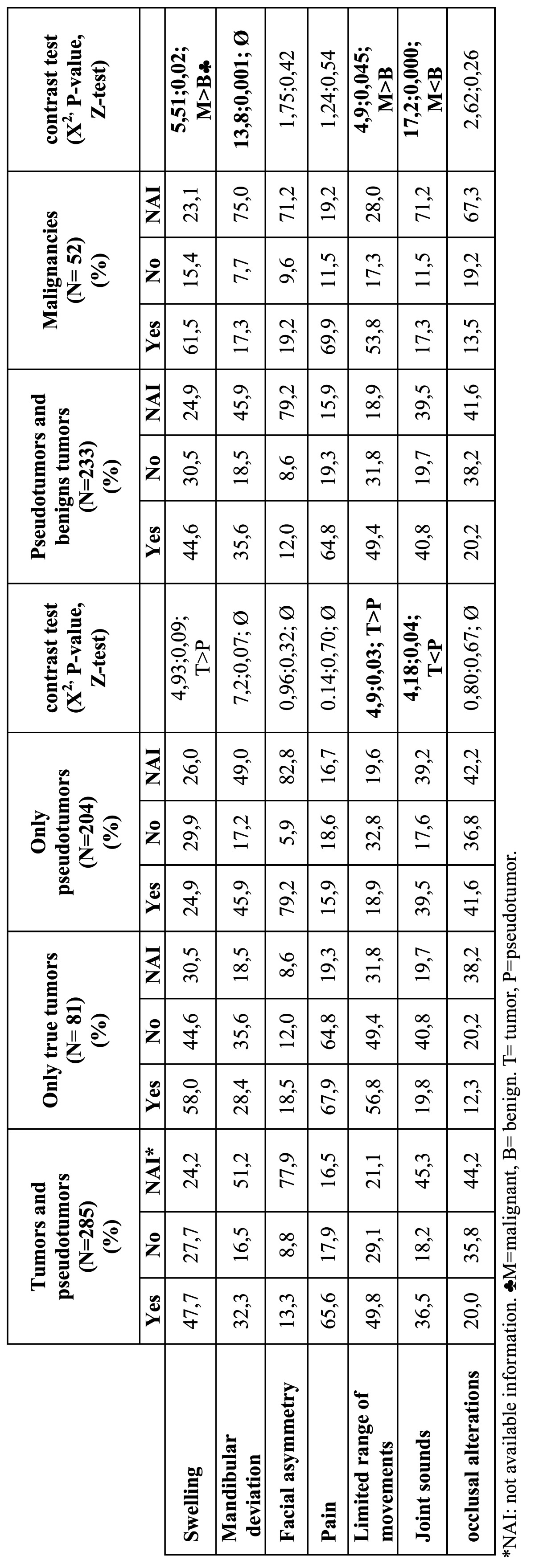
Clinical manifestations. Global Data. Comparison between tumors and pseudotumors. Comparison between benign tumors and malignancies (in percent).

**Table 2:**
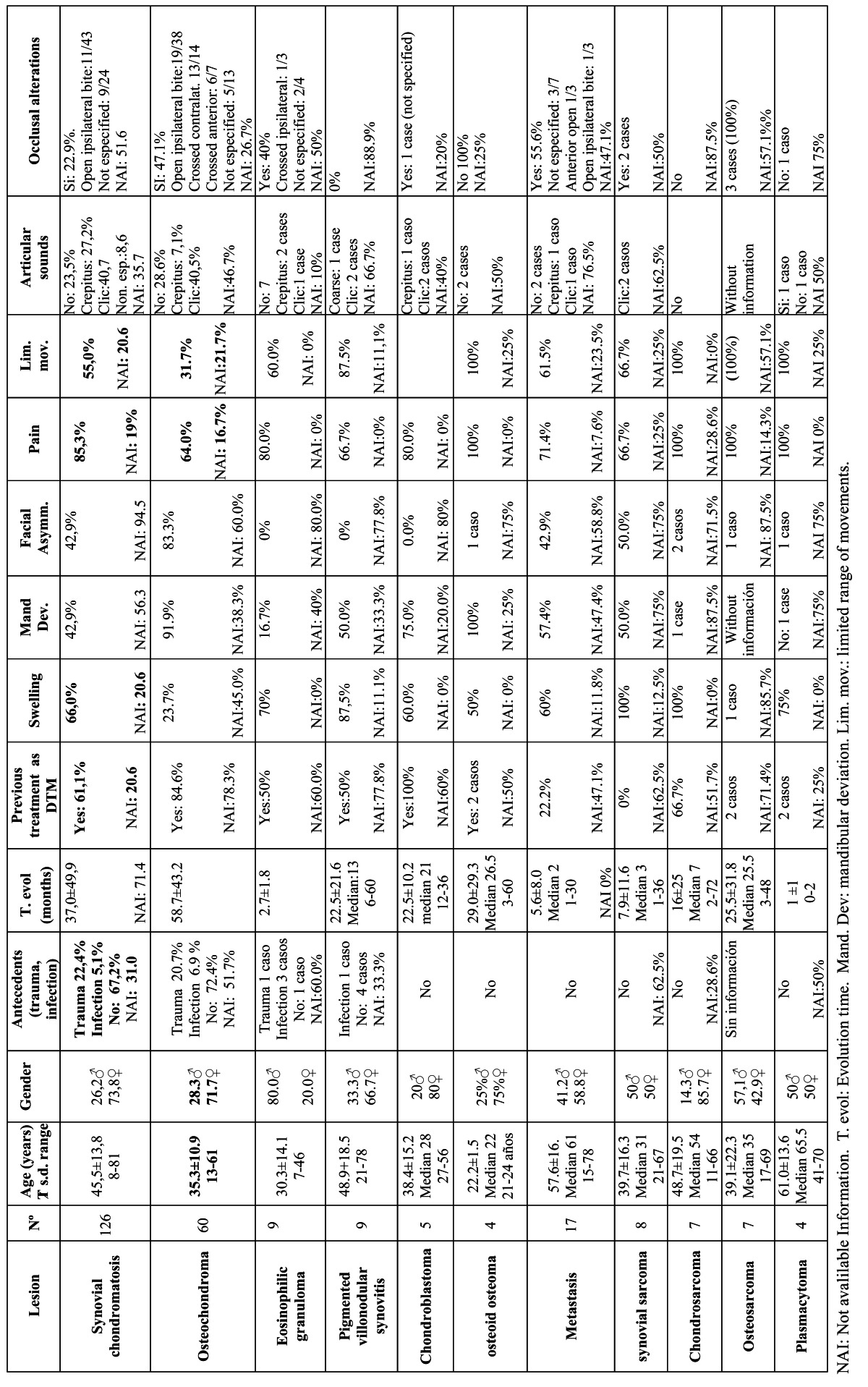
Clinical variables in tumors and pseudotumors of the TMJ.

**Table 3:**
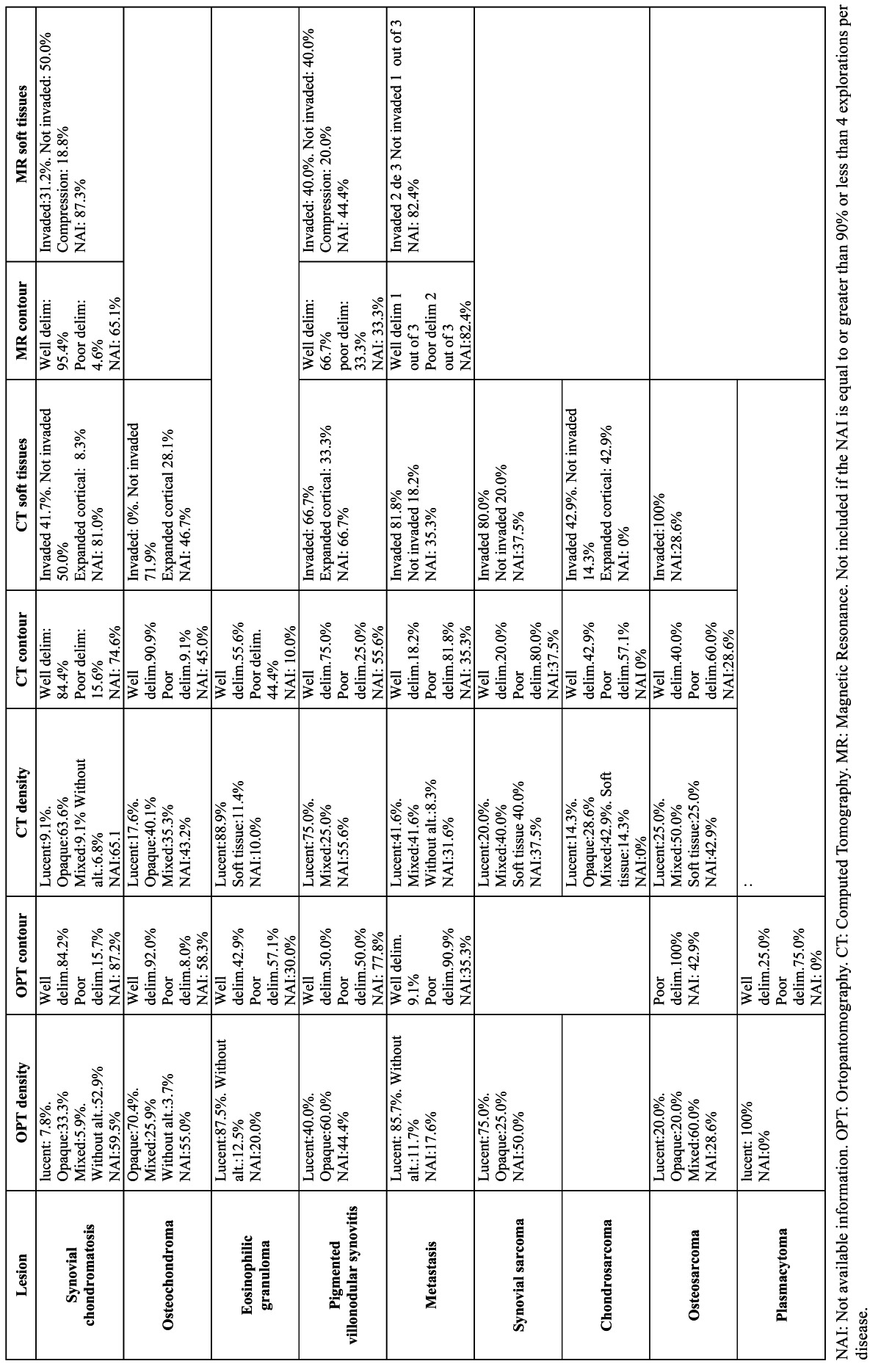
Image alterations in tumors and pseudotumors of the TMJ.

**Table 4:**
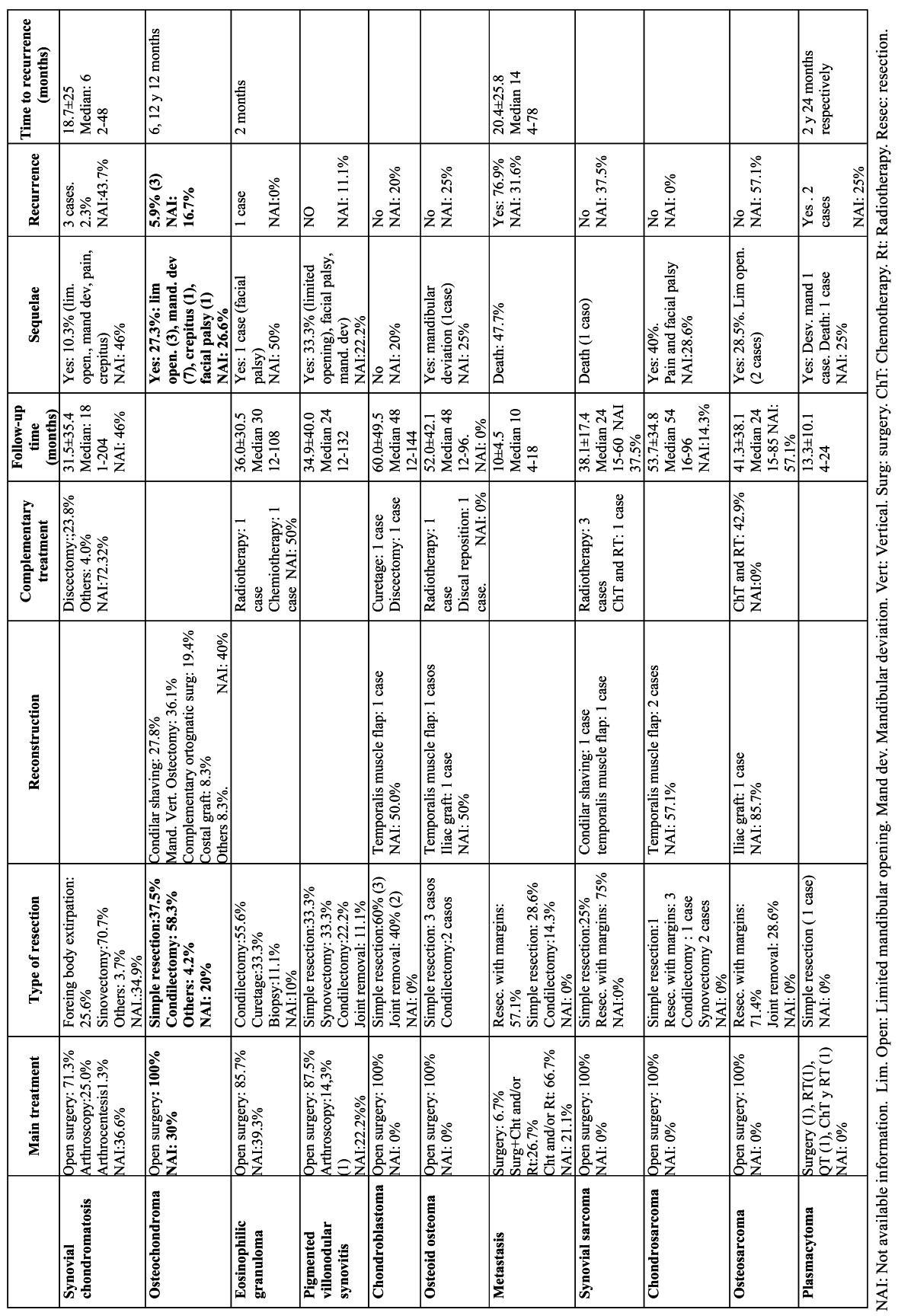
Treatment and evolution of tumors and pseudotumors of the TMJ.

## Results and Discussion

-Bibliometric data

Temporomandibular joint tumors were seen to be published in 15 of the 56 journals included in the review (26.8%). Most of them were found in the *Journal of Oral and Maxillofacial Surgery* (30.2%), *Oral Surgery Oral Medicine Oral Pathology Oral Radiology and Endodontics* (18.6%), the *International Journal of Oral and Maxillofacial Surgery* (12.6%), *Dentomaxillofacial Radiology* (11.9%), and the *British Journal of Oral and Maxillofacial Surgery* (8.4%).

We identified 285 tumors and pseudotumors of the TMJ published in 181 articles. Most corresponded to isolated cases (156 lesions, 54.7%), while the rest (129 lesions, 45.3%) were case series involving 2-14 cases each. The number of publications per year ranged from three (in 1995) to 18 (in 2007).

 Table 1 contains frequency of clinical manifestations, comparison between tumors and pseudotumors and comparison between benign tumors and malignancies. Table 2, Table 3, Table 4 contain epidemiological, clinical, diagnostic prognostic and therapeutic data about the most common tumors of the TMJ.

-Clinical data

Most of the 285 identified lesions were benign (81.8%). However, on excluding the pseudotumors (synovial chondromatosis, pigmented villonodular synovitis, eosinophilic granuloma and osteochondroma), the situation changed drastically; in effect, of the 81 tumors which could be regarded as true tumors, almost two-thirds (64.2%) were seen to be malignant.

The lesions were usually diagnosed in young individuals (mean age 42 years and 2 months ± 16 years and 2 months), though with a broad age range (4-81 years). These figures remained similar on excluding the pseudotumors from the analysis and limiting the study to the true tumors (mean age 42 years and 6 months, ± 20 years and 3 months) (range 4-78 years). Different results were obtained on comparing the age of patients with benign lesions versus those with malignant tumors. In effect, the latter were significantly older at the time of diagnosis than the patients with benign lesions (48 years and 9 months ± 19 years and 1 month versus 40 years and 6 months ± 15 years and 1 month; t=3.65, p<0.002). The recorded mean age is consistent with the 51 years reported by Bavitz et al. (12) at the time of diagnosis of malignant TMJ tumors. On excluding pseudotumors from the analysis, the difference in age between the benign and malignant tumors was found to increase, since the mean age of the patients with true benign tumors was 31 years and 4 months ± 17 years and 6 months (t=4.2, p<0.000). It is advisable to include malignant TMJ tumors in the differential diagnosis in the case of elderly patients showing a sudden onset of symptoms (13).

In the same way as TMJ derangement or dysfunction (TMJD), temporomandibular joint tumors are more frequent in females than in males (14). However, as the seriousness of the process increases, the gender difference decreases. Two-thirds of the pseudotumors and tumors were identified in women (66.3%) (female-male ratio 1.97:1). On excluding the pseudotumors and limiting the analysis to the true tumors, this female predominance was seen to decrease to 55.4%. A total of 51.7% of the true benign tumors and 57.4% of the malignant tumors were diagnosed in women. Bavitz et al. reported a similar frequency of malignant tumors in men and women (12).

**Figure 1:**
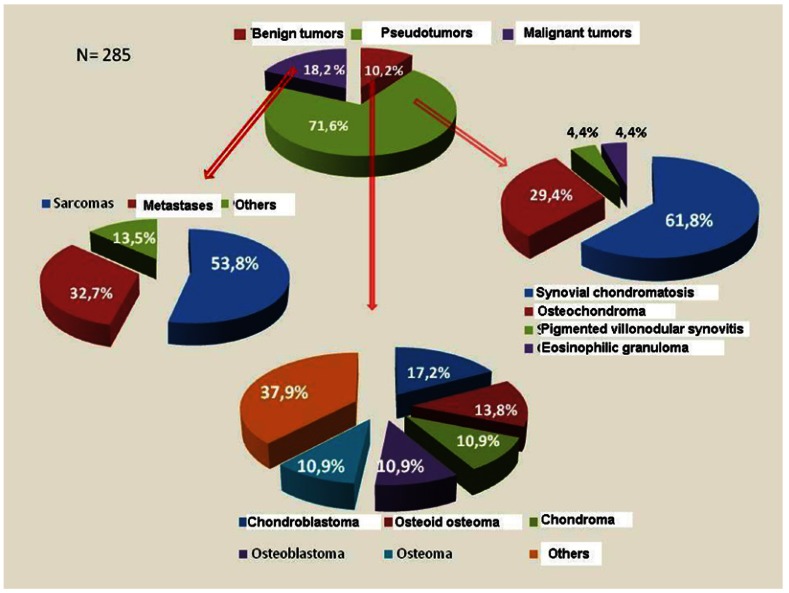
Distribution of temporomandibular joint tumors and pseudotumors.

Figure 1 shows the observed diseases and their frequency. The pseudotumors were clearly the most numerous, representing over two-thirds of the lesions (71.6%). Synovial chondromatosis accounted for almost one-half of the cases (44.2%) and 61.8% of the pseudotumors. Benign mesenchymal tumors were the most frequent true tumors, followed by sarcomas and metastases. Our findings differ from those reported by Allias et al., who found the most frequent malignant TMJ tumors to be metastases (15).

The mean time from symptoms onset to first consultation was approximately 2.5 years (30 months ± 41 months and 10 days), though this interval could not be established in 35.4% of the cases. A statistically significant difference was observed in the mean time from symptoms onset to first consultation between benign lesions (3 years ± 3 years and 9 months) and malignancies (9 months and 14 days ± 13 months and 23 days) (t=6.35; p=0.000). On analyzing only the true benign tumors, the mean time decreased slightly to 25 months and 22 days - the difference with respect to the malignant tumors remaining statistically significant (t=3.07; p=0.005).

The clinical manifestations of TMJ pseudotumors and tumors are very similar to those of TMJD, thereby frequently giving rise to errors in diagnosis and to inadequate treatment. In effect, one out of every 5 cases (19.6%) in our study initially had been diagnosed and treated as TMJD. However, the true percentage is very likely quite higher, since this information was not available in two-thirds of the cases (66.7%). The conclusions that can be drawn on analyzing the cases where the diagnostic and treatment information was available are of clinical relevance, since among the pseudotumors and benign tumors it was available in 29.2% of the cases, and of these, two-thirds (63.2%) initially had been diagnosed and treated as TMJD. In turn, in the malignant tumors the information was available in one-half of the cases, and in 48.1% the patients initially had been diagnosed and treated as TMJD.

 Table 1 shows the dichotomic clinical variables corresponding to the global tumors and to the true tumors and pseudotumors considered separately, with an analysis of the differences between them. The benign lesions and malignant tumors are likewise considered separately, with an analysis of the differences between them. Swelling presented a negative predictive value of over 90% for malignant tumors (91.2%).

The analyzed qualitative clinical variables with more than two categories and quantitative clinical variables were lesion consistency, joint sounds, clinical size and occlusal alterations. The clinical consistency of the lesion was only documented in 40 cases (13.9%); the great majority were found to be hard in response to palpation (82.5%) with no differences between pseudo-tumors and tumors (χ2=2.40; p=0.12) or between benign and malignant tumors (χ2=1.28; p=0.26).

Information on the presence or absence of joint sounds was available in 54.7% of the patients. Clicks were the most frequent sounds (40.4%), followed by crepitus (21.8%). One-third of the tumors produced no joint sounds (33.3%). Both clicks and crepitus were more common among the pseudotumors than in the tumors (χ2=16.1; p=0.003). No significant differences were recorded between malignant and benign tumors in terms of joint sounds (χ2=1.36; p=0.72), and in both cases clicks were the most frequent sound.

Occlusion alterations, one of the signs most suggestive of joint tumors together with the presence of swelling, were reported in 20.5% of the patients – this proportion being slightly greater than the 10% reported by Bavitz et al. for malignant tumors (12). In one-third of the cases there were no occlusion disorders, and in almost one-half of the cases studied (44%) no mention was made of whether such alterations were recorded or not. The most common occlusal disorders were ipsilateral posterior open bite, contralateral cross-bite and anterior cross-bite. Contralateral open bite, ipsilateral cross-bite or anterior open bite was reported in less than 2% of the cases. Ipsilateral open bite - one of the most suggestive clinical signs - was only documented in one-third of the tumors (34.0%) in which the presence of occlusal disorders was registered. Similar findings apply to contralateral cross-bite, which was only reported in 14 of the 285 tumors (4.9%). There were no significant differences in occlusion disorders between pseudotumors and tumors (χ2=0.80; p=0.67) or between benign and malignant tumors (χ2=3,9;p=0.14).

-Imaging techniques

The panoramic X-ray (P-Rx) study revealed the presence of a radiotransparency in approximately one out of every 5 patients (19.1%), a radiopacity in 13.9%, and mixed images in 6.3%. Two findings appear particularly relevant: in almost one-half of the cases (46.5%) no mention was made of whether there were P-Rx alterations or not, and in 40 cases (14.0%) such alterations were reported to be absent – this being explained by the fact that a calcium loss of 50% or more is required in order for the lesion to be radiographically visible (12)-. Radiotransparencies were significantly more frequent in the tumors (77.8%) than in the pseudotumors (22.0%) (χ2=40.9; p=0.000). In contrast, radiopacities were significantly more common in the pseudotumors (61.0%) than among the tumors (7.4%). The most common imaging finding in the benign lesions was radiopacity – the latter not being seen in any of the malignant tumors. Among the malignancies, radiotransparencies were the most frequent image (53.7%); however, it would not be correct to conclude that radiotransparencies are pathognomonic or characteristic of malignancy, since they were also reported in 11.1% of the benign tumors. Nevertheless, perhaps the most relevant observation was the fact that almost one of every 10 malignant tumors (9.3%) showed no P-Rx alterations. Radiotransparency presented a negative predictive value of 90.8% for malignant tumors.

On limiting the analysis to the true benign tumors, the radiological pattern was seen to change, with a predominance of radio-transparencies (44.8%). Nevertheless, radiotransparent images remained significantly more frequent among the malignant tumors (χ2=9.05; p=0.01). As regards lesion delimitation, P-Rx established clear differences between benign and malignant tumors, though unfortunately this information was only available in 103 of the 285 tumors (36.5%). The great majority of the benign tumors (80.3%) showed a well delimited contour, while practically the same proportion of malignancies (79.3%) exhibited a poorly delimited contour. The difference was statistically significant (χ2=31.37; p=0.000). A poorly defined contour in the P-Rx study presented a negative predictive value of 90.0% for malignant tumors.

Computed tomography (CT) was available in 159 cases (55.8%). The frequency of radiopaque images in the CT study was greater in the case of benign lesions than in the malignant tumors (19.2% versus 3.7%), while mixed and soft tissue patterns were more frequent among the latter (29.6% and 14.8% versus 11.1% and 3.8%, respectively) (χ2=36.8; p=0.000). The data referred to lesion contour were practically analogous to those recorded in the P-Rx study – 81.2% of the benign lesions showing a well delimited contour, while 76.9% of the malignant tumors presented a poorly delimited contour (χ2=41.76; p=0.000). Of note is the observation that approximately 20% of the patients with malignant tumors presented a lesion with well delimited contours in both the P-Rx study and the CT scan. The invasion of adjacent soft tissues as evidenced by CT was significantly more frequent in the malignant tumors (85.7%) than in the benign lesions (29.9%)(χ2=28.7; p=0.000). The negative predictive value of the invasion of soft tissues was 90.3%.

-Treatment and outcome

Surgery was the treatment prescribed in most of the tumors. A preauricular approach was used in almost one-half of the patients (48.0%), though the true percentage is probably considerably greater, since information referred to the surgical approach was not available in over a third of the cases (36.5%). Arthroscopic treatment was used in 7.3% of the cases, and practically all of these corresponded to synovial chondromatosis (95%). In 58 benign lesions (24.9%) no mention was made of the type of treatment provided. In the cases where such information was available (176 patients, 75.5%), the most frequent treatment was found to be total or partial synovectomy (34.7%). This is because synovectomy is the technique of choice in synovial chondromatosis, which accounted for 44.2% of all the tumors. The rest of the treatments in decreasing order of frequency were simple tumor resection (23.3%), resection with safety margins (9.8%), and simple foreign body removal (7.4%), which is also one of the options in synovial chondromatosis. The most common surgical procedure in the case of malignant disease was tumor resection with margins (46.2%), followed by simple tumor resection (20.5%). In comparison, in the benign tumors, the most frequent treatment was simple resection (53.6%), followed by condylectomy (17.9%). In 31 tumors, use was made of radiotherapy or chemotherapy, or a combination of both (13, 6 and 12 patients, respectively). All but one of these cases (osteoid osteoma) were malignant.

The mean duration of patient follow-up was 30.5±30.1 months, with a median of 22.5 months. In 84 cases (29.5%) information referred to the duration of follow-up was not available. A little over one-half of the patients (54.2%) suffered no sequelae following treatment, while 18.4% experienced some problems. In the rest of the cases no mention was made of whether there were sequelae or not. Among the 54 cases in which treatment complications were reported, the most common problems were mandibular deviation (27.8%), limited range of movement (16.7%), facial palsy (7.4%) and crepitus and pain (5.5% in each case). Fifteen patients (27.8%) died as a result of the neoplasm (all corresponding to malignant tumors).

**Figure 2:**
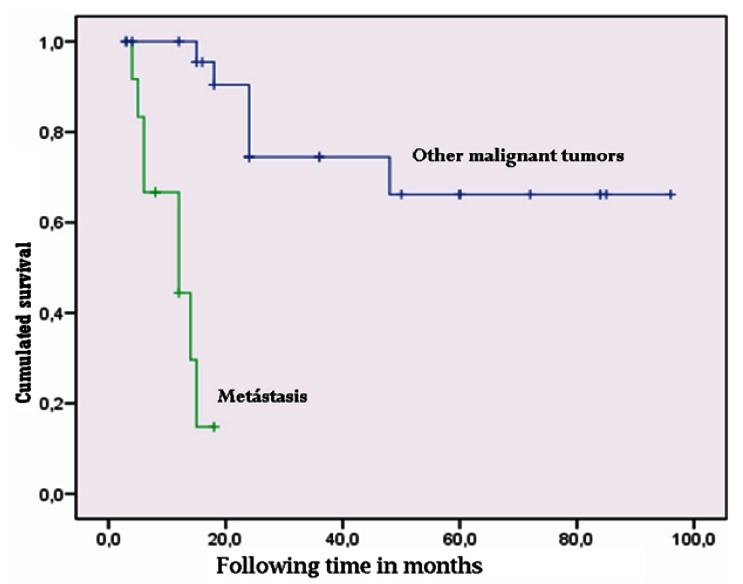
Comparison of survival in temporomandibular joint metastatic disease versus other malignancies of the temporomandibular joint.

**Figure 3:**
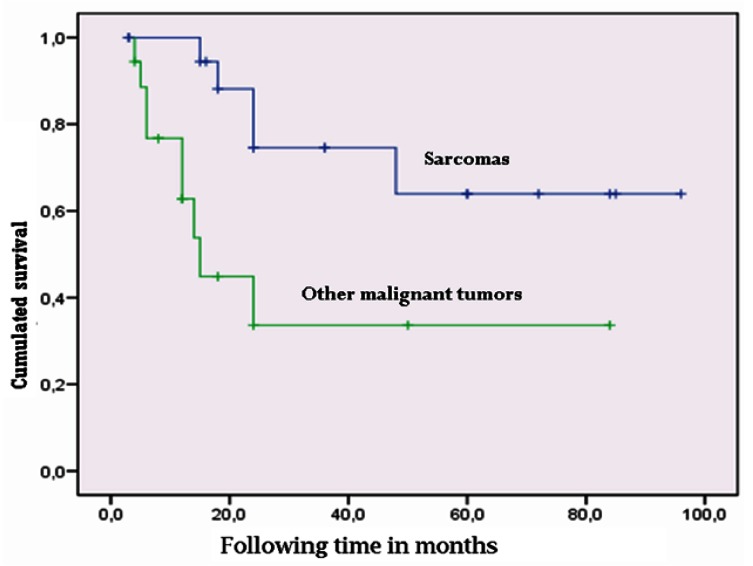
Comparison of survival in temporomandibular joint sarcomas versus other malignancies of the temporomandibular joint.

Kaplan-Meier survival analysis of the 39 malignant tumors followed-up on for a period of 3-96 months yielded an estimated mean survival of approximately 5 years (57.9 months; standard error (SE) 7.6, 95% confidence interval (95%CI) 43.0-72.7 months). On differentiating between metastasis and the rest of malignant tumors, we found metastatic disease to present a survival of about one year (11.4 months; 95%CI 8.6-14.1 months), while the rest of the malignant tumors showed a mean survival of 6 years (72.9 months; SE 7.8, 95%CI 57.6-88.3). Comparison based on the Mantel-Cox log-rank test showed the difference to be statistically significant (χ2=24.4; p=0.000) (Fig. 2). In contrast, sarcomas (the most frequent malignant tumors of the TMJ in our study) showed longer survival than the non-sarcomatous malignancies (plasmacytomas, lymphomas, metastases, etc.), with a mean of 69.7 months (SE 9.5, 95%CI 51.1-88.3 months) versus 40.2±9.7 months in the case of the rest of malignant tumors. The log-rank test showed the difference to be statistically significant (χ2=46; p=0.03) (Fig. 3). Survival after 5 years among the TMJ sarcoma patients (65%) was similar to that reported by Yamaguchi et al. for oral and maxillofacial sarcomas (61%) (16).

Tumor relapse was documented in 26 cases (9.1%), of which 10 corresponded to benign lesions (4.3%) and 16 to malignant tumors (30.8%). On comparing proportions, and as expected, relapse was significantly more common in the case of malignant disease than in patients with benign tumors (χ2=36.5; p=0.000). No significant differences were observed in terms of the frequency of relapse between true benign tumors and pseudotumors (χ2=0.24; p=0.63). Of the mentioned 10 benign tumor relapses, three were osteochondromas, three corresponded to synovial chondromatosis, two were eosinophilic granulomas, one was diagnosed as osteoblastoma and another corresponded to osteoma. Regarding the malignant tumors, of the 17 relapses, 10 corresponded to metastasis, while four were sarcomas (angiosarcoma, synovial sarcoma and leiomyosarcoma), two plasmacytomas and one Hodgkin lymphoma. Curiously, the chondrosarcomas and osteosarcomas - representing 26% of the malignant tumors and 50% of the sarcomas - showed no relapses in our study. In contrast, Yamaguchi et al. reported relapse or metastasis in 5 out of 9 orofacial osteosarcomas (16), while Oliveira et al. described recurrences in 9 out of 20 chondrosarcomas of the TMJ (17).

In conclusion, tumors of the temporomandibular joint are characteristically found in young adults, and because of the scant specificity of the symptoms, many of them are initially diagnosed and treated as temporomandibular joint derangement. Pain, swelling and the limitation of joint movements are the most frequent clinical manifestations. Other more specific alterations such as facial asymmetry or occlusal disorders, are less common. Many tumors show no radiological alterations in the P-Rx study. Radiotransparency, and especially a poorly defined tumor contour, are suggestive of malignancy. Treatment usually involves surgery, and relapse is observed in 10% of the cases – particularly among malignant tumors.
